# Micro‐Architected Lithium Cobalt Oxide

**DOI:** 10.1002/advs.202513312

**Published:** 2025-10-08

**Authors:** Yuchun Sun, Julia R. Greer

**Affiliations:** ^1^ Division of Engineering and Applied Science California Institute of Technology Pasadena CA 91125 USA; ^2^ Kavli Nanoscience Institute California Institute of Technology Pasadena CA 91125 USA

**Keywords:** 3D electrodes, 3D printing, additive manufacturing, lithium cobalt oxide, micro‐architected electrodes

## Abstract

Advancements in additive manufacturing (AM) enable the precise engineering of micro‐architected electrodes with enhanced electrochemical and mechanical properties. Existing AM approaches for fabricating lithium‐ion battery cathodes rely on extrusion‐based direct ink‐writing, which is usually limited to 150–200 µm resolution, or vat photopolymerization (VP) 3D printing with metal salt solution, which is limited in material choices due to the complicated photoresin design and printing parameter optimization. A gel infusion AM technique is introduced to fabricate micro‐architected cathodes, using lithium cobalt oxide (LCO) as a model prototype, which utilizes VP 3D printing with a “blank” photoresin to circumvent these limitations. The synthesized micro‐architected LCO electrodes are free‐standing and binder‐free, with beam diameters below 50 µm and tunable microstructure and mechanical resilience. The nanoindentation modulus of differently oriented LCO grains varies between 148.4 and 286.6 GPa, with no grain boundary weakening. This electrode gives a reversible capacity of 122–142 mAh g^−1^ (11.3–13.2 mAh cm^−2^) up to a current density of 28 mA g^−1^ (2.6 mA cm^−2^). This method is adaptable for a broad range of cathode materials, which opens a promising pathway to fabricate micro‐architected electrodes with fully controllable form factors, versatile material choices, and micro‐sized resolution for future energy storage solutions.

## Graphical abstract

This work introduces a gel infusion additive manufacturing technique for the fabrication of micro‐architected lithium‐ion battery cathodes, exemplified with LiCoO_2_. The resulting electrodes are free‐standing and binder‐free, with tunable microstructure and independent structural control across multiple length scales. Minimizing the need for photoresin formulation and printing parameter optimization, this technique simplifies 3D cathode fabrication and extends applicability across diverse chemistries.

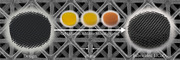

## 1 Introduction

Additive manufacturing (AM) offers an efficient approach to construct self‐supporting, 3D electrode architectures, which have attracted substantial interest for electrochemical applications as their tailored architectures can potentially enhance ion transport and structural stability, enabling both high energy and power densities.^[^ ^1^, ^2^ ^]^ Among the diverse range of AM techniques, the extrusion‐based direct ink‐writing (DIW) stands out as an early technique to fabricate 3D architected electrode materials. This technique utilizes a blend of electrode active material particles, polymers, and solvents to produce a viscous ink. The ink is extruded through a nozzle as a viscous ribbon, which solidifies in the process of building a prescribed 3D structure. Sun et al. demonstrated the utility of DIW in the fabrication of Li_4_Ti_5_O_12_ and LiFePO_4_ 3D electrodes for rechargeable lithium‐ion batteries (LIB), and Gao et al. developed a self‐standing sulfur/carbon composite electrode using DIW.^[^ ^3^, ^4^ ^]^ Extrusion‐based methods have several drawbacks, for example the requirement for the inks to be viscous and the need for overhanging parts support, which complicates the post‐print processing and renders them incapable of producing features with micron‐ and sub‐micron resolution while maintaining adequate pattern fidelity. These limitations cause most ink‐written electrodes to have the form factors of vertically stacked 2D‐patterned layers, whose aspect ratios are constrained by the maximum number of layers that can be self‐supporting when vertically stacked.^[^ ^5^ ^]^ The electrode feature sizes are predominantly determined by the extrusion nozzle diameter, designed to accommodate the rheological properties of the viscous inks, with thinner nozzles enabling the production of finer features but being more susceptible to clogging. Typical line thicknesses of ink‐written microlattice electrodes are around 150–200 µm, as demonstrated for sulfur/carbon composite electrodes and holey graphene oxide electrodes.^[^ ^4^, ^6^ ^]^ These limitations in 3D geometries and resolution of DIW hinder its potential for accessing a broad parameter space of architectural design and for optimizing Li‐ion transport in the electrode or in the electrolyte.

Vat‐photopolymerization (VP)‐enabled additive manufacturing is a promising technique that allows for the fabrication of high‐resolution 3D architectures compared with extrusion‐based 3D printing and offers significant flexibility and control of attainable geometries and printing parameters. This method utilizes a photo‐polymerizable liquid resin, which can be uniquely synthesized, and is subsequently cured with UV light according to a layer‐by‐layer algorithm to produce the computed or designed 3D structures. Despite its higher resolution compared to DIW, VP faces more challenges with incorporating functional materials into the polymer 3D structure. A common strategy to produce the desired materials shaped into a prescribed architecture using VP has been to incorporate the material micro or nanoparticles into the initial photoresin blend, then to subject the particle‐containing printed resins to thermal processing to remove the polymer.^[^ ^7^ ^]^ The presence of particles in the photoresin prior to printing often leads to adverse effects ‐ locally, like inhomogeneous polymerization and diffused gelation profiles caused by their absorption and scattering of UV light, and globally, i.e., non‐uniform feature density that results from particle sedimentation in the resin suspension during long (several hours) prints. To overcome these issues, Yee et al. first dissolved the metal nitrate salts as electrode material precursors in an aqueous photoresin, then 3D printed the desired shape using a VP 3D printer and synthesized the electrode material during post‐print thermal processing.^[^ ^8^ ^]^ This method opened a pathway for high‐resolution AM of 3D electrode materials; its generalization to a broader range of materials is hindered by the need to individually customize photoresin composition and optimize 3D printing parameters for each material. The solubility of electrode material precursors constrains the choice of solvent‐photoinitiator‐UV blocker combinations, with their unique interactions with UV light further increasing the complexity of photoresin design for high‐resolution electrode printing. The micro‐architected LiCoO_2_ and LiNi_0.33_Mn_0.33_Co_0.33_O_2_ electrodes fabricated using this technique still have beam diameters of 100–300 µm.^[^ ^8^, ^9^ ^]^

We introduce a VP‐based gel infusion AM technique capable of creating 3D micro‐architected electrodes with a resolution of ≈45 µm and demonstrate its feasibility on the fabrication of LiCoO_2_ (LCO) 3D microlattices.^[^ ^10^ ^]^ This process begins with VP 3D printing of prescribed 3D geometry with feature size of 120 µm using a customized photoresin to first create a blank organogel microlattice. After solvent exchange that replaces the original organic solvent with water, the 3D printed hydrogel microlattice is infused with LCO precursors, LiNO_3_ and Co(NO_3_)_2_, and then subjected to a high‐temperature thermal treatment process during which the polymers combust, and the metal ions react with oxygen to form a metal oxide polycrystal. The final product is a uniformly shrunk solid LCO replica of the original geometry, with ≈45 µm‐diameter beams, ≈55% smaller than what is possible using VP process capabilities today.^[^ ^8^ ^]^ This electrode is then placed in in a coin cell against lithium metal anode with liquid electrolyte and subjected to electrochemical cycling, which exhibits an average specific discharge capacity of 135 mAh g^−1^ in the first two cycles at C/40, and its discharge capacity remains above 130 mAh g^−1^ at increasing C‐rates up to C/2. Material characterization reveals that exploring the parameter space of processing conditions offers tunability in the microstructure and mechanical resilience of LCO. This method utilizes VP's advantage of high resolution while simplifying photoresin design, making it adaptable for a wide range of electrode materials.

## 2 Fabrication Procedure

**Figure 1:**
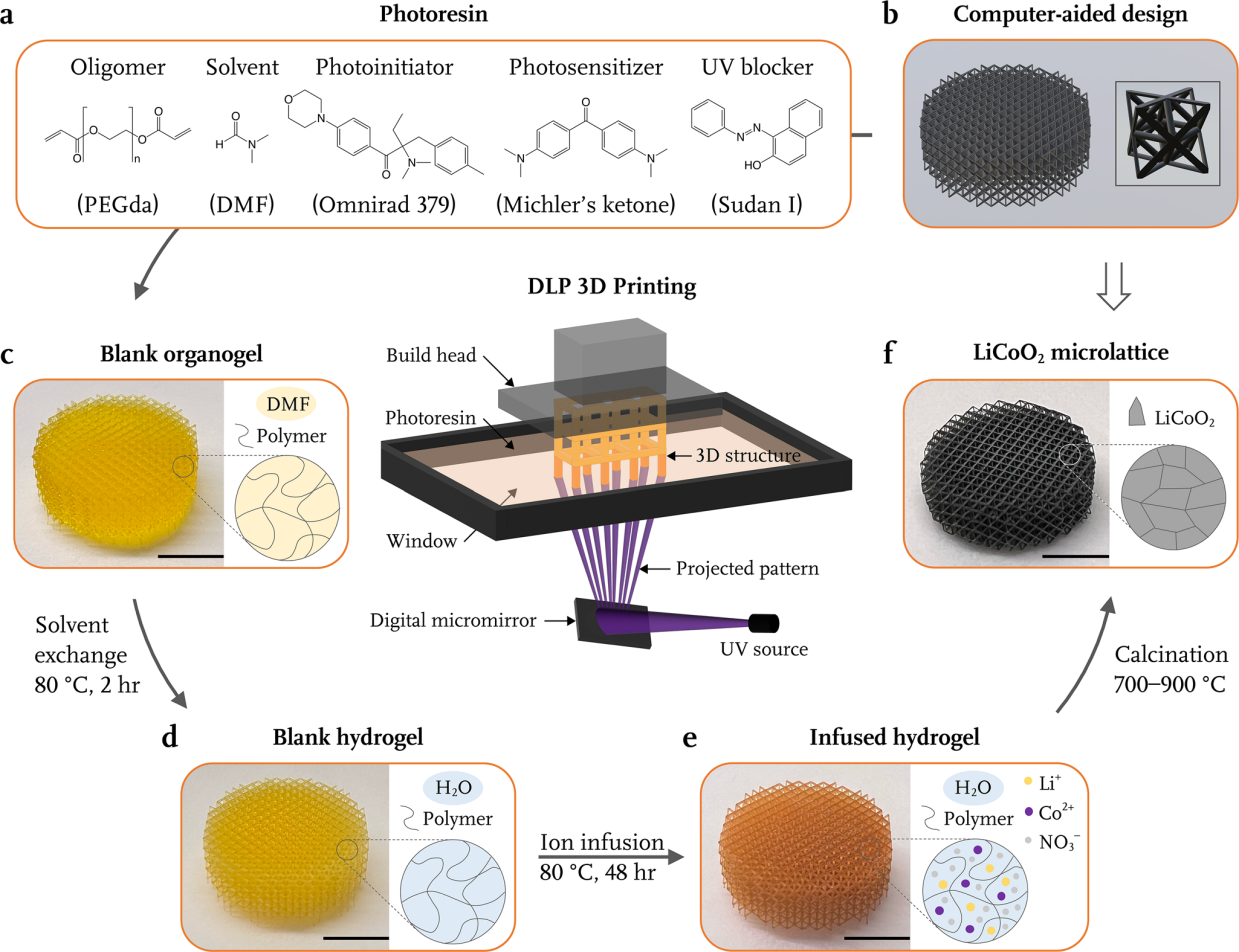
Vat polymerization (VP) additive manufacturing of 3D micro‐architected electrodes. Schematic of digital light processing (DLP) 3D printing (center). a) Composition of the photoresin for VP 3D printing. b) Computer‐aided design (CAD) of an example octet truss 3D lattice, with unit cell shown in the inset. Optical images and schematics of c) printed blank organogel after soaking in *N,N*‐dimethylformamide (DMF), d) blank hydrogel after solvent exchange with water, e) hydrogel infused with Li^+^ and Co^2+^ ions, and f) solid lithium cobalt oxide (LCO) microlattice after thermal treatment that synthesizes LCO and burns off the organic content. Scale bars are 5 mm for (c), (d) and (e); 2 mm for (f).

**Figure** **1** illustrates the gel infusion AM process to manufacture 3D micro‐architected electrodes. First, a digital light processing (DLP) 3D printer with a 385 nm UV laser is used to produce the desired light pattern via a digital micromirror array, passing it through a transparent window to expose and harden the photoresin according to the pattern one layer at a time. Figure 1a shows the composition of the customized acrylate‐based photoresin, and Tables S1 and S2 (Supporting Information) contain additional composition and printing parameters.

Figure 1b shows a computer‐aided design (CAD) of the chosen periodic octet truss lattice that is a beam‐based cellular solid with horizontal and 45° angled beams; a single unit cell of the octet truss is shown in the inset. Figure 1c shows the 3D organogel lattice with 120 µm‐diameter, 707 µm‐long beams printed using printing parameters of 25 µm XY pixel size and 10 µm layer thickness, which is subsequently subjected to solvent exchange in water at 80 °C for 2 h, transforming it into a hydrogel (Figure 1d). The absence of electrode material powders or their precursors in the photoresin prevents undesired UV absorption and scattering during printing. The hydrogel lattice is then infused with metal ions in an aqueous solution with 2M LiNO_3_ and 2M Co(NO_3_)_2_ at 80 °C (Figure 1e). Once the ion infusion process reaches equilibrium, indicated by a stable gel mass after 48 h, the ion‐infused hydrogel part is calcined in a tube furnace at a temperature of 400 °C to form a sintered metal oxide polycrystal via simultaneous oxygen diffusion, metal oxidation, and grain nucleation. Calcination is conducted at 0.01 atm with air flowing at 5 mL min^−1^ at a temperature ramp of 0.25 °C min^−1^ from room temperature to 400 °C, followed by an isothermal hold for 5 h (Figure S1a, Supporting Information). After the reaction‐completing isothermal hold, the air flow is switched to 14 mL min^−1^ Ar flow at 1 atm, and the temperature further ramps at 2 °C min^−1^ up to 700 °C to induce LCO sintering, before cooling to room temperature at 1 °C min^−1^. Calcination and sintering allow for LCO nucleation, densification and grain growth, forming a solid LCO phase within the originally designed form factor, with ≈60% isotropically shrunken linear dimensions (Figure 1f). Figure S2 (Supporting Information) shows additional sample geometries: cubic lattices with various beam diameters and unit cell sizes, which demonstrate the versatility and architectural design freedom offered by this gel infusion AM process.

## 3 Structural, Microstructural, and Mechanical Characterization

**Figure 2:**
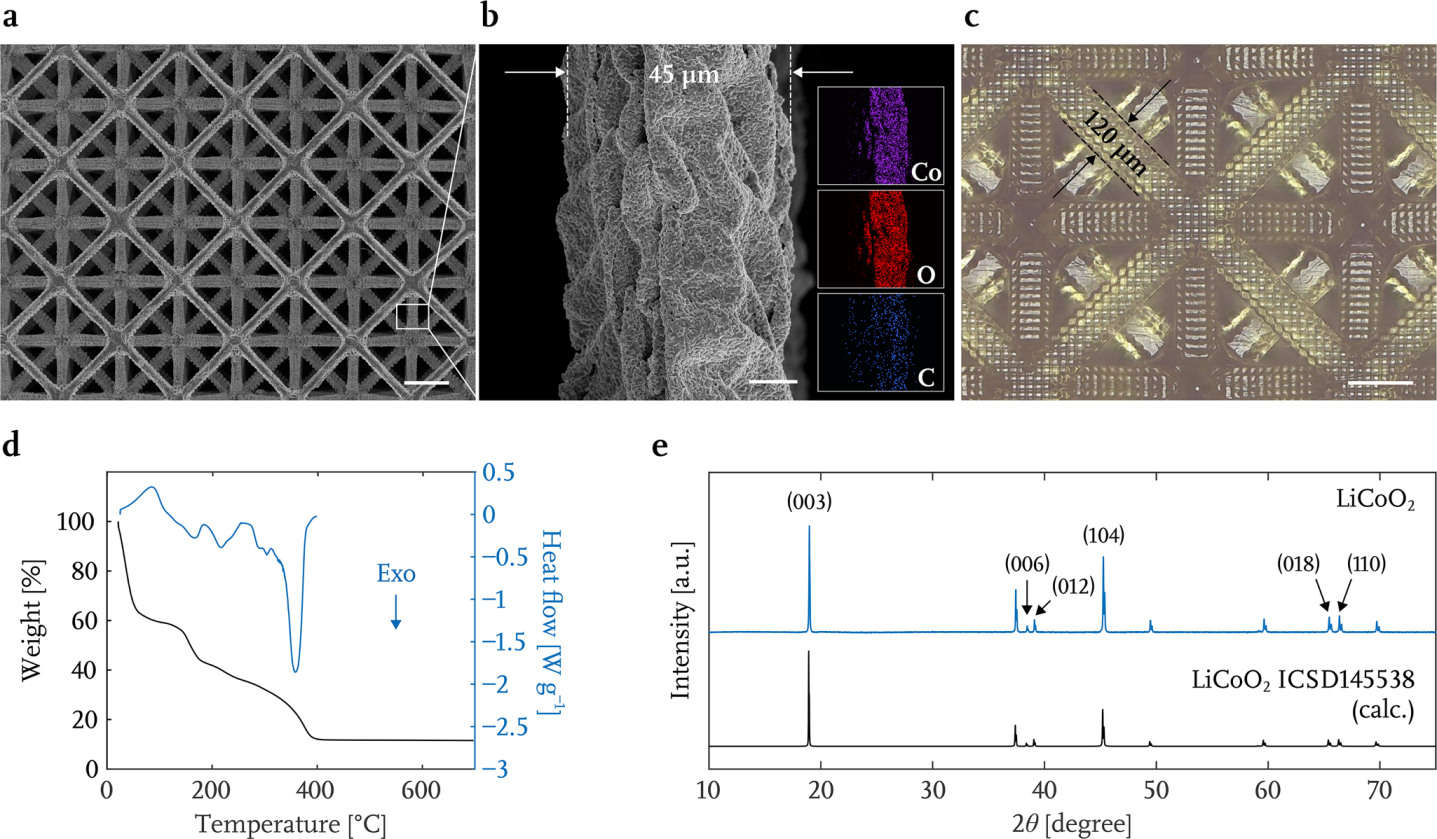
a) Scanning electron microscopy (SEM) image of the top view of a representative 3D micro‐architected LCO lattice. b) A zoomed‐in SEM image of an individual beam within the LCO lattice, with energy dispersive X‐ray spectroscopy (EDS) elemental maps for each element shown in the insets. c) Optical image of the top view of a 3D printed gel. d) Thermogravimetric analysis (TGA) and differential scanning calorimetry (DSC) profiles of Li^+^‐ and Co^2+^‐infused gels during the air heating. e) X‐ray powder diffraction (XRD) pattern of the additively manufactured LCO sintered under 1 atm Ar at 700 °C compared with a reference from ICSD. Scale bars are 200 µm for (a) and (c); 10 µm for (b).

**Figure** **2**a contains a top‐view scanning electron microscopy (SEM) image of a representative LCO microlattice that reveals its periodicity and interconnectivity, with a zoomed‐in view of a typical 45 µm diameter beam shown in Figure 2b, which also shows the pixel‐type surface roughness characteristic of DLP 3D printing. This periodic surface roughness is due to the square pixels and constant layer thickness of the printing, which is clearly seen on the printed gel as shown in Figure 2c. Elemental maps generated by energy dispersive X‐ray spectroscopy (EDS) show a uniform distribution of Co, O and C atoms; this technique is not capable of detecting Li (Figure 2b, insets). Inductively coupled plasma mass spectroscopy (ICP‐MS) analysis confirms a Li:Co molar ratio of 1.01:1 and reveals 5wt.% of carbon generated by the incomplete oxidation of the decomposed polymers (Table S3, Supporting Information). We find that this residual carbon mainly resides at the LCO grain boundaries (Figure S3, Supporting Information) and likely serves to enhance the electrical conductivity of the electrode by providing a conductive percolating path throughout the LCO structure. The desired presence of carbon in the additively manufactured LCO mirrors the practice of conventional slurry cathode fabrication where carbon‐based materials are intentionally introduced as electron conducting additives.^[^ ^11^ ^]^

Thermogravimetric analysis (TGA) and differential scanning calorimetry (DSC) analysis of the infused gel shown in Figure 2d reveal the endothermic dehydration below 150 °C, followed by the exothermic decomposition and combustion reactions between polymers, metal salts and O_2_ in air over the temperature range of 150 to 400 °C, with an overall reaction as 2LiNO_3_ + 2Co(NO_3_)_2_ + 2kC_26_H_46_O_13_ + (62k‐7)O_2_ → 2LiCoO_2_ + 52kCO_2_ + 46kH_2_O + 3N_2_, where k is the molar ratio of poly(ethylene glycol) diacrylate oligomers (*M*_n_ = 575) to Li and Co ions. The slow temperature ramping rate of 0.25 °C min^−1^ and low O_2_ concentration in the reaction atmosphere at a reduced pressure were chosen for calcination to limit the combustion reaction and the outgassing rate to enable the LCO microlattices to retain their structural integrity. The unbounded water weighs 40% of the total mass of the infused gel, and the final LCO lattice retains 13% of its original mass, based on the TGA analysis.

X‐ray powder diffraction (XRD) confirms the formed LCO crystals to be a hexagonal α‐NaFeO_2_ layered structure in the trigonal $R\bar{3}m$ space group, shown in Figure 2e, against a reference from Inorganic Crystal Structure Database (ICSD). The clear splitting of (006)/(012) and (018)/(110) diffraction peaks suggest well‐layered hexagonal crystal structure.^[^ ^8^ ^]^

**Figure 3:**
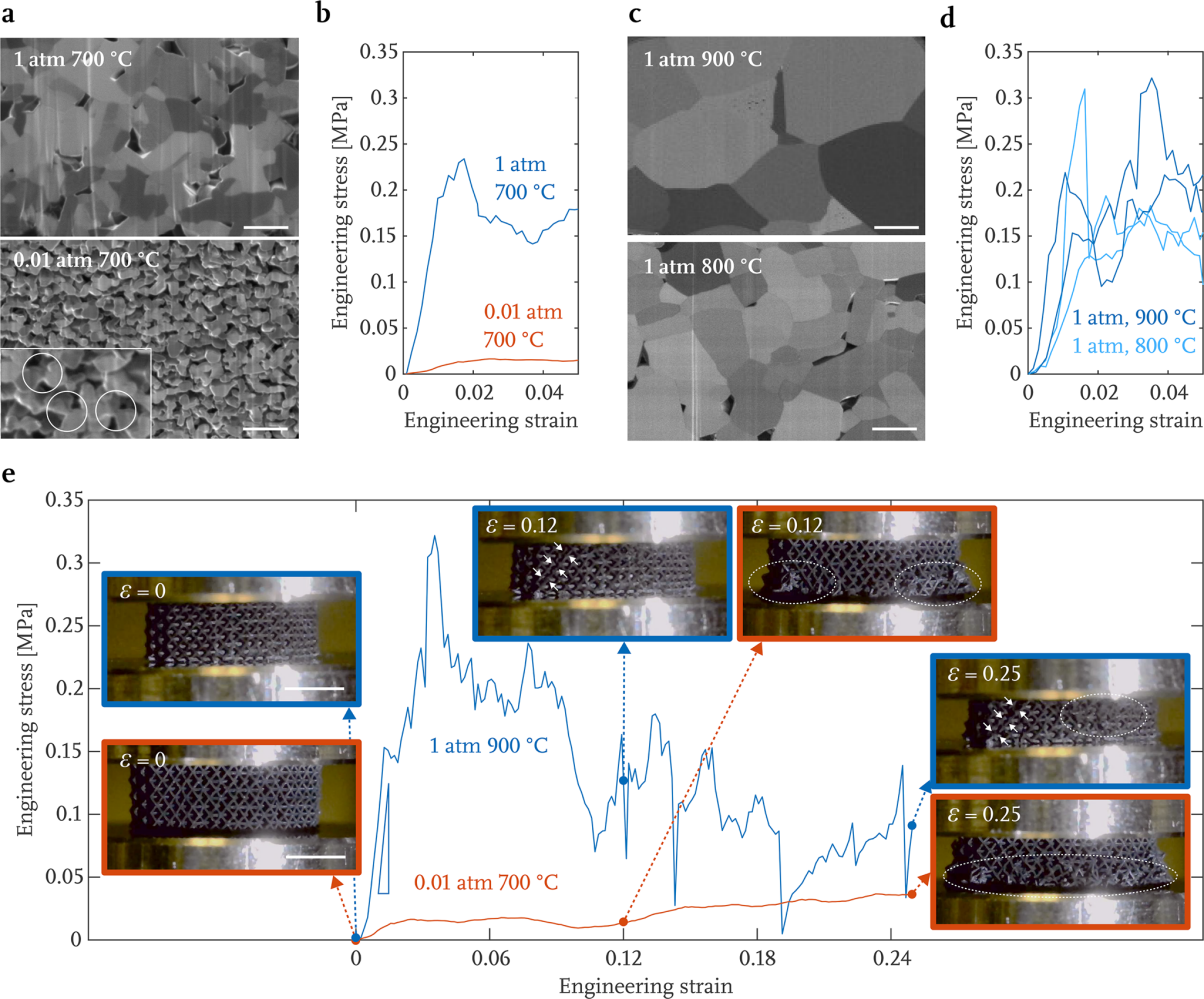
a) Focused ion beam (FIB)‐acquired ion channeling contrast images of the beam cross‐sections in LCO microlattices sintered under 1 atm in Ar (top) and 0.01 atm in air (bottom) at 700 °C, with necking between LCO particles evident in the latter highlighted in the inset. b) Engineering stress–strain data collected during a quasi‐static compression of the same LCO microlattices. c) FIB‐acquired ion channeling contrast images of the beam cross‐sections in LCO microlattices sintered under 1 atm in Ar at 900 and 800 °C. d) Engineering stress–strain data collected during a quasi‐static compression of the same LCO microlattices. e) Time‐lapse engineering stress–strain data for representative samples sintered at 900 °C under 1 atm in Ar (top data set) and at 700 °C under 0.01 atm in air (bottom data set), with strain‐correlated snapshots of each sample also shown. The slope of the top data set corresponds to a stiffness of 19 MPa. Scale bars are 1 µm for (a); 2 µm for (c); 2 mm for (e).

Exploring the parameter space of the thermal treatment process indicates that the in‐air combustion of the infused gel at a reduced pressure of 0.01 atm produces a porous nanocrystalline LCO microstructure, with a resulting grain size of ≈500 nm after sintering under 1 atm Ar at 700 °C, as illustrated in the top ion channeling contrast image in **Figure** **3**a. The porosity of LCO in the microlattice is approximately 24% (Figure S4d, Supporting Information). To tailor the LCO microstructure, we explored different atmospheres and temperatures during the sintering stage, while keeping the same conditions during LCO synthesis below 400 °C. Our results indicate that when the air atmosphere is maintained at 0.01 atm during sintering, the LCO lattice experiences an isotropic linear shrinkage to ≈53% of the original gel size (Figure S5, Supporting Information) with a concomitant reduction in the grain size to ≈100 nm (Figure 3a) and a greater porosity of 33% (Figure S4e, Supporting Information), similar to those observed in Yee et al.^[^ ^8^ ^]^ This reduced shrinkage of ≈47% in the overall dimensions of 3D lattices, compared to ≈60% for samples sintered under 1 atm Ar, can be explained by the higher LCO porosity caused by limited densification. The LCO grains produced under these conditions are characterized by rounded geometries, more free surfaces, and fewer grain boundaries, with most crystallites fused via sintering‐induced necking (Figure 3a, inset).

To investigate the mechanical behavior of LCO samples with the various microstructures generated by different sintering atmospheres, we conducted uniaxial compression experiments at a quasi‐static strain rate of 10^−3^ s^−1^ using a dynamic mechanical analyzer. We first examined the effect of pressure; Figure 3b shows the typical engineering stress–strain data for the samples heated to 700 °C under different pressures, calculated by normalizing the load–displacement data by the cross‐sectional footprint area of the overall samples and the initial height. The observed stress–strain behavior indicates that the LCO samples sintered under 1 atm are an order of magnitude stronger than those sintered at 0.01 atm, exhibiting a peak compressive stress of 234 kPa. This disparity in strength can be attributed to the 0.01 atm sintered samples having a greater fraction of grain boundaries that terminate at a free surface, which creates a distribution of stress concentrations along the surface, with weaker ones serving as failure initiation sites under global compression.^[^ ^12^ ^]^ The combination of fewer surface stress concentrations and the inter‐granular frictional interlocking in the 1 atm‐sintered LCO contribute to its greater structural integrity.

We then explored the impact of sintering temperature on the microstructure of additively manufactured LCO and its effect on the mechanical behavior. The sintering temperatures of 700–900 °C for LCO correspond to the homologous temperatures of approximately 0.6–0.8, consistent with the solid‐state sintering practices.^[^ ^13^, ^14^ ^]^ Figure 3c shows that grain size increased with sintering temperature at a constant pressure of 1 atm under Ar: samples sintered at 800 °C produce ≈2 µm grains and those sintered at 900 °C are twice as large. Figure 3d shows the stress–strain data of the quasistatic uniaxial compression experiments for samples sintered at these different temperatures and reveals that they are virtually indistinguishable, which implies that this variation in grain size is not the key factor in governing the overall mechanical strengths of LCO microlattices.

The crystal structure of LCO sintered under various temperatures and pressures discussed above are confirmed with XRD analysis (Figure S6, Supporting Information). The 1 atm‐sintered samples consistently produce LCO with high purity. While 0.01 atm sintering at 700 °C gives pure LCO microlattices, a higher temperature of 900 °C under the same reduced pressure introduces undesired crystalline Co_3_O_4_ and CoO. The presence of these phases likely stems from a substantial loss of Li during high temperature sintering under reduced pressure, confirmed by a decreased Li to Co molar ratio of 0.79:1 in ICP‐MS analysis.

Figure 3e contains a plot of two representative engineering stress–strain responses with the effective axial strain of 25% for LCO microlattices with different microstructures formed through labeled annealing conditions, with corresponding video snapshots of strain‐correlated micrographs revealing their vastly different mechanical signatures. The 1 atm‐900 °C microlattice has a loading stiffness of 19 MPa and attains a yield stress of 153 kPa, a peak compressive stress of 322 kPa at 3.5% strain, and an average plateau stress of 204 kPa between 4.0% and 9.2% strain, as well as exhibits stochastic deformation, with multiple load drops throughout the compression, which typically correspond to local structural instabilities, either through micro‐cracking or buckling. The stress–strain response of the 0.01 atm‐700 °C sample shows yield at a low stress of 13 kPa followed by an extensive and smooth stress plateau that extends from 13 kPa at a strain of 1.6% to 36 kPa at the maximum compressive strain of 25%, which suggests homogeneous material and structural deformation. Early node fracture, likely due to the imperfect initial contact between the LCO microlattice and the indenter tip, occurred in both samples during incipient compression, and the full contact was established at ≈0.5% strain. Combined with the corresponding video frames, these results convey further differences in their failure mechanisms. In the 0.01 atm‐700 °C sample, multiple nodes at the bottom of the microlattice fractured simultaneously at a stress below 18 kPa, as captured by the video snapshot at 12% strain. Up to 25% strain, local instabilities facilitate efficient load redistribution and lead to the self‐similar, homogeneous deformation throughout the lower part of the microlattice. Although the similar local node fracturing is also present in the 1 atm‐900 °C microlattice, this sample mainly shows discrete response of layer‐by‐layer collapse up to 25% strain, exhibiting its continuously weaker load bearing capacity accompanied by densely spaced discrete stress drops during individual instabilities. The video snapshot at 12% strain highlights an individual layer collapse, accompanied by a rapid stress reduction from 163 to 65 kPa before reloading. These results indicate that the LCO sample sintered at the reduced pressure of 0.01 atm, whose microstructure is micro‐porous and ultra‐fine‐grained, is substantially weaker than the one that was sintered at the higher pressure and contains coherent, micro‐sized grains, as indicated by an order of magnitude difference in their yield strengths and plateau stresses up to 9.2% strain. While the uniaxial compression experiments were conducted in air, it is important to recognize that actual operation of the electrodes usually occur in other environments. When the micro‐architected LCO is cycled in liquid electrolyte, the formation of cathode electrolyte interface (CEI) film may slightly alter its fracture behavior under the quasi‐static loading, as the electrolyte may quickly fill the microcracks and induce CEI formation, but whether crack propagation will be facilitated or inhibited by CEI formation needs to be further investigated.^[^ ^15^ ^]^ If the micro‐architected electrode is filled with polymer electrolyte, the overall system becomes a solid composite where the polymer will contribute significantly to the overall mechanical strength, and the mismatch in stiffness and interfacial architecture between the LCO and the polymer phases may redirect cracks and dissipate energy at the interfaces, resulting in synergistic strengthening and toughening of both phases.^[^ ^16^ ^]^

To probe the mechanical properties of the additively manufactured LCO material, we conducted in situ nanoindentation experiments in an SEM chamber on a representative beam cross‐section within the LCO microlattice sintered in Ar at 900 °C under 1 atm. To perform the nanoindentation, the LCO lattice was first mounted in an epoxy puck and then progressively polished to reveal the grain maps within the beams, as shown in Figure S4a (Supporting Information). The nanoindentation experiments were performed on multiple grains and grain boundaries that were mapped out in the SEM, under displacement rate control of 5 nm s^−1^ using a diamond Berkovich indenter tip (Figure S7a, Supporting Information) and a continuous stiffness measurement (CSM) setting that allows to acquire contact stiffness throughout the indentation, calculated by Equation (1).^[^ ^17^ ^]^

**Figure 4:**
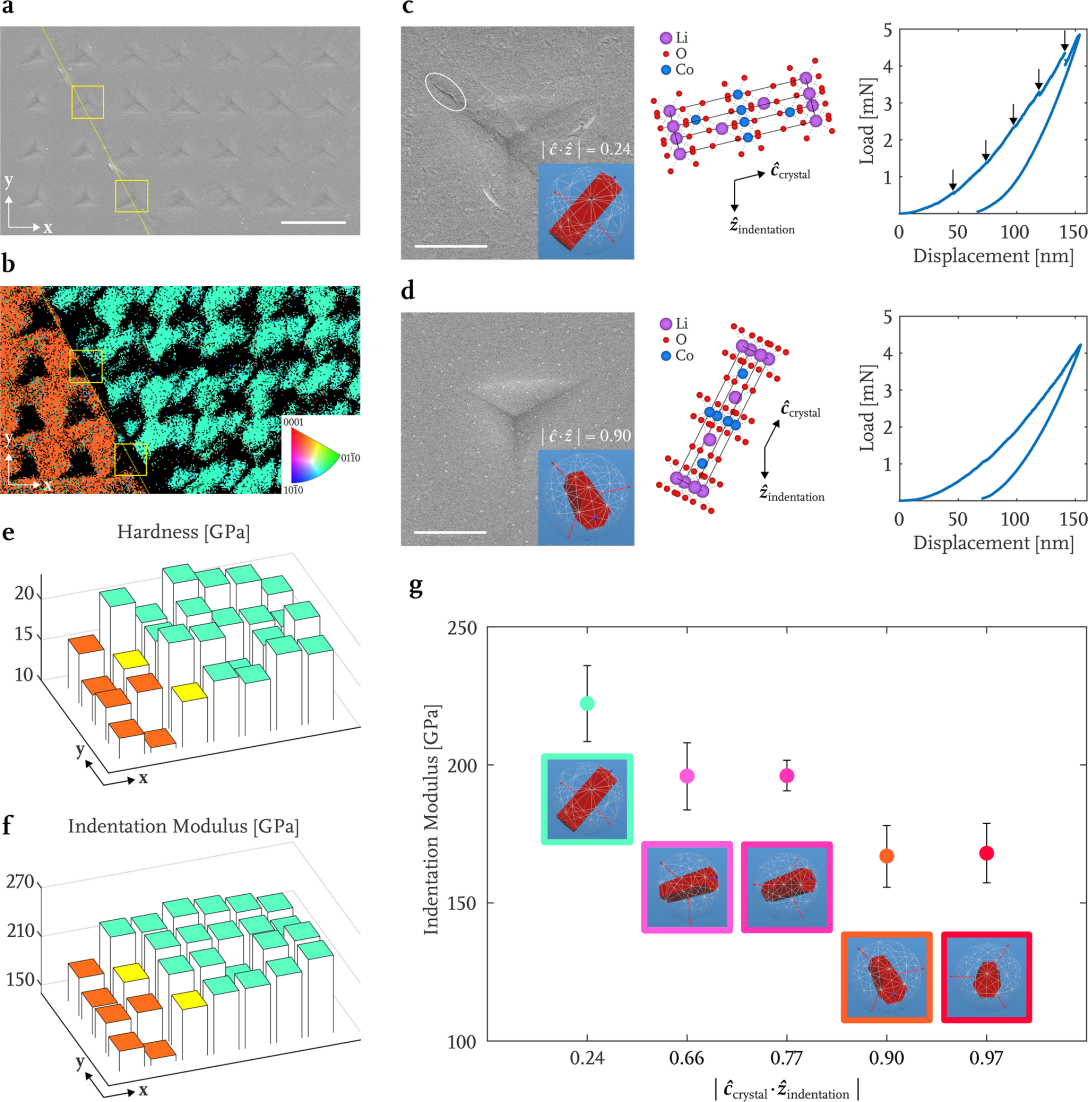
a) SEM image of the nanoindentation array mapped over the chosen sample area of a polished cross‐section of a typical beam within the 3D micro‐architected LCO sample. b) The corresponding electron backscatter diffraction (EBSD) image. Representative indentation impressions, the LCO crystallographic orientation quantified by ∣ ***ĉ***_crystal_· ***ẑ***_indentation_∣ from a view along the indentation direction (insets), the respective LCO unit cell from a view perpendicular to the indentation direction, and the corresponding load–displacement data for c) ∣ ***ĉ*** · ***ẑ*** ∣ = 0.24 and d) ∣ ***ĉ*** · ***ẑ*** ∣ = 0.90. e) Hardness and f) modulus maps of the same sample area. g) Indentation modulus as a function of LCO crystal orientation, with inset schematics showing the crystal orientation of each LCO grain from a view along the indentation direction. Scale bars are 2 µm for (a); 500 nm for (c) and (d).

**Figure** **4**a shows a representative nanoindentation array that spans two adjacent LCO grains, with two indentation marks, highlighted in yellow, located on the grain boundary. Figure 4b displays the complementary post‐indentation electron backscatter diffraction (EBSD) map of the same area that shows grain orientation in the out‐of‐plane direction. The inverse pole figure that shows colors as a function of crystallographic orientation is provided in the inset. Crystallographic orientations of each grain were determined using EBSD, extracted from areas away from the indentation marks, and the alignment between the crystallographic orientation of the grain and the applied load is quantified as the absolute value of the dot product between unit vectors in these directions, denoted as ∣ ***ĉ***_crystal_· ***ẑ***_indentation_∣.

Figures 4c–d show SEM images of two residual indentation impressions, with the insets showing the LCO crystallographic orientation from a view along the indentation direction, the respective LCO unit cell orientation from a view perpendicular to the indentation direction, and the corresponding load–displacement data. The indentation direction for the grain shown in Figure 4c is nearly perpendicular to [0001] crystal orientation (∣ ***ĉ*** · ***ẑ*** ∣ = 0.24); the load–displacement signature for this grain contains multiple discrete load drops throughout the indentation, highlighted by arrows, which correspond to the formation of several LCO basal plane cracks along the indentation marks. Its hardness as a function of contact depth, calculated using Equation (5) and shown in Figure S7c (Supporting Information), exhibits notable discontinuities, with a 12% decrease around 140 nm indentation depth, which corresponds to a pronounced load drop in the load–displacement data. The indentation modulus, calculated using Equation (4) and shown in Figure S7d (Supporting Information), decreases with depth, with a ≈60 GPa difference between the indentation depths of 40 and 150 nm. Figure 4d shows the indentation into a grain whose [0001] crystal direction is virtually aligned with the direction of applied load (∣ ***ĉ*** · ***ẑ*** ∣ = 0.90) and exhibits a smooth load–displacement signature, with no apparent load drops throughout the 150 nm total indentation depth. This orientation exhibits a steady hardness and a relatively stable indentation modulus with a smaller difference of ≈30 GPa between the indentation depths of 40 and 150 nm (Figures S7f–g, Supporting Information), compared to the LCO grain with ∣ ***ĉ*** · ***ẑ*** ∣ = 0.24.

Figures 4e–f show the hardnesses and indentation moduli mapped over the region in Figures 4a–b that contains two LCO grains and a grain boundary. There are two indentations performed precisely on the grain boundary, colored in yellow. These maps point to an unambiguous dependency of the elastic properties of LCO on its grain orientation, as well as to the rules‐of‐mixtures grain boundary behavior, which suggests a lack of grain boundary weakening or softening, as may have been observed in Li‐containing oxide ceramics.^[^ ^18^ ^]^ Figure 4g summarizes the indentation moduli for five distinct LCO crystal orientations, and reveals a systematic reduction in modulus as the crystallographic c‐axis of LCO approaches the indentation direction, from 222.2 ± 13.8 GPa for ∣ ***ĉ*** · ***ẑ*** ∣ = 0.24 to 168.1 ± 10.8 GPa for ∣ ***ĉ*** · ***ẑ*** ∣ = 0.97. Across all individual measurements, the indentation modulus varies between 148.4 and 286.6 GPa.

Anisotropic elasticity in LCO has been previously reported. For example, Wu et al. calculated Young's modulus for LCO with the energy strain approach utilizing two different packages:^[^ ^19^ ^]^ Vienna ab intio Simulation Package (VASP) gave E_X_ = 321.05 GPa / E_Z_ = 212.83 GPa and Cambridge Serial Total Energy Package (CASTEP) gave E_X_ = 290.06 GPa / E_Z_ = 177.25 GPa, where X and Z correspond to [11$\bar{2}$0] and [0001] directions, respectively. Qu et al. conducted nanoindentation experiments on 21 differently oriented LCO grains and used the Oliver‐Pharr method to calculate the Young's modulus of 151–236 GPa.^[^ ^20^ ^]^ In their method, the contact stiffness was calculated from the unloading data after reaching a maximum indentation load of 2 mN, corresponding to a typical indentation depth of around 100 nm. The Oliver‐Pharr method has the underlying assumption of isotropic elasticity, which renders it inaccurate for the highly anisotropic materials like LCO.^[^ ^21^, ^22^, ^23^ ^]^ The accuracy of these elastic constants' calculations also requires the material to not fracture during loading because crack closure during the unloading segment will contribute to additional measured displacement. This is particularly relevant for nanoindentation into the brittle LCO, where the inhomogeneous stress distribution under the indenter tip can lead to stress concentrations, and ultimately to fracture. Our experiments revealed such local failures to occur at the shallow depths of ≈50 nm for the grains whose crystallographic orientations, ∣ ***ĉ*** · ***ẑ*** ∣, were close to 0; we observed more stable behavior during nanoindentation into the grains with orientations, ∣ ***ĉ*** · ***ẑ*** ∣, close to 1. The commonly observed early failure in the grains with certain orientations suggests a potential underestimation of LCO's modulus calculated from the unloading data in nanoindentation experiments, especially at maximum depths greater than 50 nm. This underestimation is evident based on the results in this work where nanoindentation was conducted using the CSM mode, which allowed us to determine Young's modulus more precisely using Equations (1)–(4), and which reveals a significant decrease in indentation modulus with depth, consistent with our observations of cracking shown in Figure 4c.

## 4 Electrochemical Characterization

**Figure 5:**
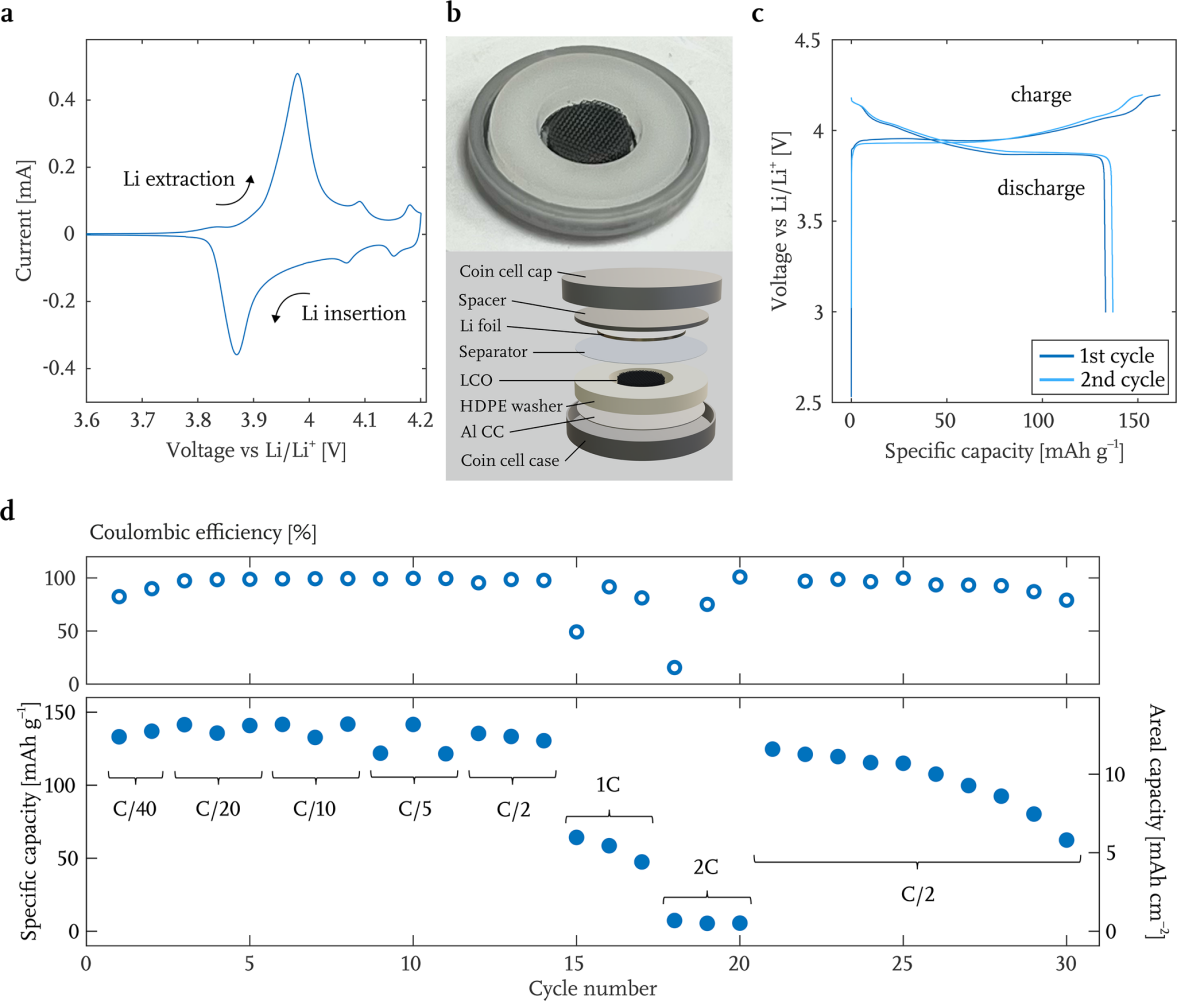
Electrochemical performance of additively manufactured LCO sintered at 700 °C under 1 atm Ar. a) Cyclic voltammogram (CV) of LCO microlattices pulverized into a slurry. b) Optical image of the LCO microlattice surrounded by a high density polyethylene (HDPE) washer on aluminum current collector (Al CC) in a CR2032 coin cell case (top) and schematic image of coin cell components (bottom). c) First and second charge–discharge data during galvanostatic cycling of the LCO microlattice at C/40. d) Coulombic efficiency (top) and discharge specific and areal capacities (bottom) of the LCO microlattice cycled between 3.0 and 4.2 V at different C‐rates.

We chose the micro‐architected LCO lattice sintered at 700 °C under 1 atm Ar to serve as a representative sample for electrochemical analysis. To decouple the intrinsic material electrochemical property of this additively manufactured LCO from its 3D structure, cyclic voltammetry (CV) was performed between 3.0 and 4.2 V on the slurry electrode fabricated from pulverized LCO microlattices, with a thickness of 20 µm (**Figure** **5**a. There is a major pair of peaks at 3.98/3.87 V, which corresponds to the transformation of Li_x_CoO_2_ through a solid state reaction process between two O3 hexagonal phases as x is in the region of x = 1–0.7, concomitant with a drastic change in electronic properties.^[^ ^24^, ^25^ ^]^ The two pairs of peaks at 4.09/4.07 V and 4.18/4.15 V signal the distortion to the monoclinic phase near x = 0.5 and the transformation to a disordered O3 hexagonal phase for compositions with further decrease in Li content.^[^ ^26^ ^]^

We assembled the half‐cell in a CR2032 coin cell with LCO microlattice against Li metal, with LCO mass loading of 93 mg cm^−2^. Compared with conventional slurry electrodes, this additively manufactured electrode eliminates the need for using polymer binders, while naturally incorporating about 5% of carbon in the structure as a residual of the calcination reaction.^[^ ^27^ ^]^ We included the mass of carbon in the calculation of LCO mass loading and specific capacities. The overall height and diameter of LCO lattice are 1.80 and 5.10 mm, respectively, and a high density polyethylene (HDPE) washer with a thickness of 1.78 mm and an inner diameter of 7.95 mm is used to centrally position the 3D lattice and to support the compressive stress within the sample generated by the coin cell assembly. Figure 5b contains an optical image of the LCO microlattice in a washer on an aluminum foil current collector (top) and a schematic of all coin cell components (bottom). We chose the washer to be slightly shorter than the lattice to guarantee effective contact between the LCO lattice and the aluminum current collector (Al CC) as the LCO lattice is pre‐compressed to a low axial strain of 1.1%, which is too low to cause any mechanical damage as previously shown in Figure 3b.

Figures 5c–d illustrate the cycling performance of the micro‐architected LCO lattice between 3.0 and 4.2 V at various current densities. The charge‐discharge curves of the first two galvanostatic cycles at 3.5 mA g^−1^, corresponding to a C‐rate of C/40, give 133 and 137 mAh g^−1^ of reversible capacity, with 82.4% and 89.9% of coulombic efficiency, respectively (Figure 5c). The C‐rates are calculated based on a 140 mAh g^−1^ practical capacity of LCO, which is approximately half of its theoretical capacity of 274 mAh g^−1^. The discharge capacities and the corresponding coulombic efficiencies at systematically increasing C‐rates from C/40 to 2C, followed by ten cycles at C/2, are plotted in Figure 5d. The corresponding potential curves are shown in Figure S8. C/40, C/20, C/10 and C/5 were cycled at a constant current (CC) charging and discharging, giving a stable reversible capacity of 122–142 mAh g^−1^ (11.3–13.2 mAh cm^−2^). We observed the coulombic efficiency to gradually increase from 82.4% to 98.7% in the first five cycles, and to remain at >99% until the current density increased to above C/5. For the C/2, 1C and 2C cycles, we used constant current constant voltage (CC‐CV) charging with 0.02 mA cutoff and constant current discharging. The discharge capacity remains above 130 mAh g^−1^ during C/2 and drops abruptly to 47–64 mAh g^−1^ for 1C and 5–7 mAh g^−1^ for 2C, with a concomitant reduction in the coulombic efficiencies: 95.4% at C/2, 49.2% at 1C and 15.6% at 2C for the first cycle at each rate. The capacity recovers to 125 mAh g^−1^ when the cycling rate returns to C/2, followed by a gradual capacity fade to 115 mAh g^−1^ over the next five cycles and an abrupt drop to 62 mAh g^−1^ in the subsequent five cycles. During cycling, Li is plated and stripped on the Li metal foil only in the area not covered by the HDPE washer (Figure S9, Supporting Information), rendering the effective areal current density for Li metal anode at a rate of 2C to be around 10.7 mA cm^−2^. Without Li metal surface modification or electrolyte additives, this cycling condition can rapidly lead to mossy and dendritic Li surface morphology, deteriorating the full cell performance.^[^ ^28^ ^]^ Using a high‐rate anode to match the high areal capacity of LCO microlattice may further enhance the long‐term cycling performance of the full cell.^[^ ^29^ ^]^

We invested substantial effort into developing this AM process to minimize the beam diameter of the 3D micro‐architected LCO lattices to 45 µm to reduce the Li‐ion diffusion path within the electrode. These slender beams significantly improved the discharge capacity at C/5, with an average of three cycles to be 128 mAh g^−1^, compared to the previous work by Yee et al., which reported 94 mAh g^−1^ for similar LCO cubic microlattices with 100 µm beam diameters.^[^ ^8^ ^]^ The potential for further reducing the beam size to improve capacities at rates higher than C/5 needs to be balanced against the potential trade‐off with mechanical resilience and electrical conductivity of the electrodes. Enhancing the electrical conductivity could involve optimizing the gas composition, pressure and flow rate during LCO synthesis and sintering to increase carbon content in the final structure. Our choice of an octet truss lattice consisting of beams with high aspect ratio of ≈5.8 demonstrates the capability of this AM technique to create 3D architected electrodes with intricate geometries. For applications as battery electrodes, future designs should aim for 3D structures with higher relative densities but similar minimum feature size, which could give higher areal and volumetric energy densities and potentially further enhance the overall mechanical strength of the electrode.

## 5 Outlook

**Figure 6:**
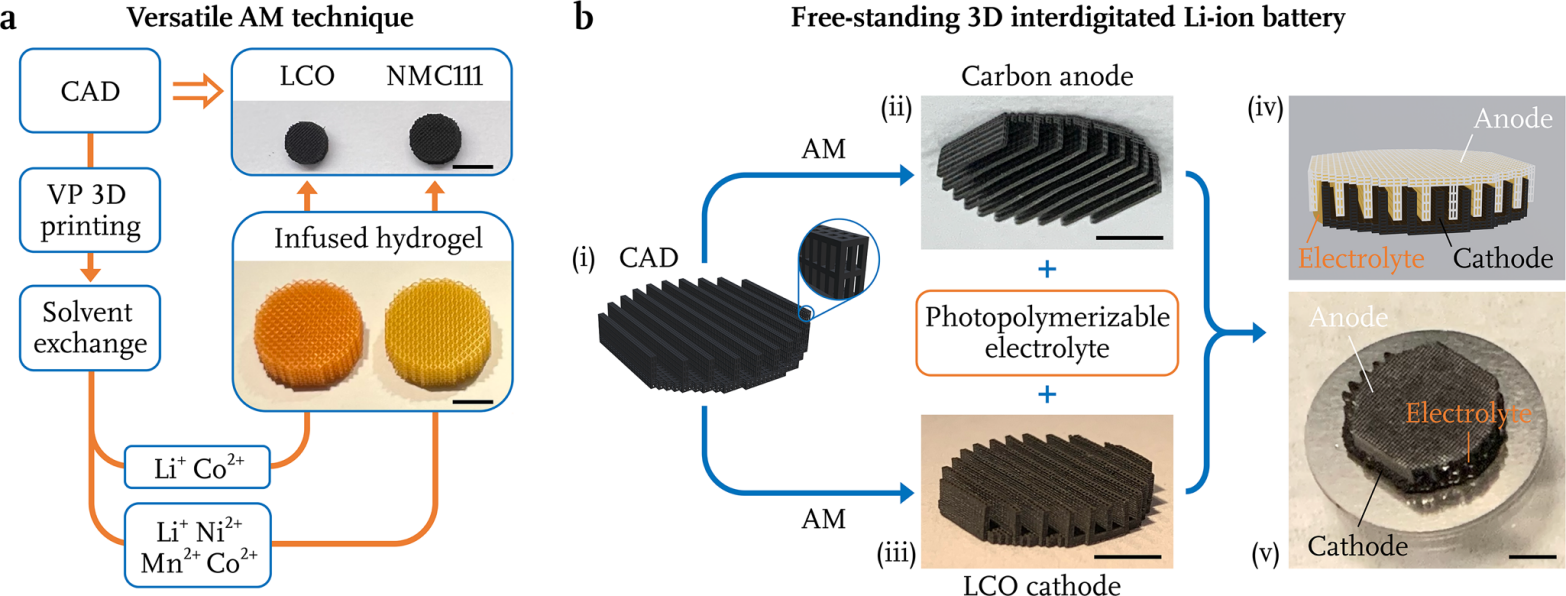
a) Fabrication of 3D micro‐architected LCO and NMC111 via parallel and virtually identical additive manufacturing processes. b) Free‐standing 3D interdigitated Li‐ion battery. i) CAD of a 3D electrode with interdigitated‐plate configuration. Optical images of ii) carbon anode and iii) LCO cathode fabricated into the corresponding structure. iv) Schematic and v) optical image of the assembled 3D battery. Scale bars are 5 mm for (a); 3 mm for (b(ii), (iii) and (v)).

The described gel infusion AM technique simplifies the creation of 3D micro‐architected cathode materials by eliminating the need for extensive photoresin design and printing parameter optimization, typically required in VP 3D printing. The LCO fabrication process showcased here is adaptable for a broad range of materials and is particularly suitable for the fabrication of two main categories of LIB cathodes: 1) lithium transition metal oxides, i.e. LiMO_2_ (M = Co, Ni, Mn, etc.) or LiM_2_O_4_ (M = Mn, etc.) type compounds, and 2) lithium metal phosphates, LiMPO_4_ (M = Fe, Mn, Ni, Co, etc.).^[^ ^30^ ^]^ This method also holds promise for mixed lithium metal oxides, such as LiNi_x_Mn_y_Co_1‐x‐y_O_2_ with tunable transition metal stoichiometry.^[^ ^31^ ^]^ **Figure** **6**a highlights the versatility of this AM technique by presenting the parallel fabrication processes of micro‐architected LCO and LiNi_0.33_Mn_0.33_Co_0.33_O_2_ (NMC111). They follow a similar initial process involving computer‐aided 3D structure design, high‐resolution VP printing of the blank organogel and solvent exchange into a blank hydrogel. The only divergence in the fabrication processes arises at the infusion of hydrogel blanks with distinct metal precursors, followed by the synthesis of each target material through a similar calcination process. Switching the infusion solution to 2 M LiNO_3_ with 0.67 M Ni(NO_3_)_2_, Mn(NO_3_)_2_ and Co(NO_3_)_2_, we demonstrated a straightforward fabrication of 3D architected NMC111 microlattices with 45 µm beam diameters (Figure S10a, Supporting Information). EDS element mapping and ion channeling contrast image of its beam cross‐section reveal a uniform distribution of O, Ni, Mn, and Co atoms within lattice beams and a grain size of ≈100 nm, with its crystal structure confirmed via XRD (Figures S10b–d, Supporting Information). To apply this AM technique across a more diverse range of other oxide and phosphate‐type cathode materials, the extensively studied sol–gel methods can often provide a robust starting point, as they could guide the selection of suitable precursors for infusion and help in determining the optimal conditions for the thermal synthesis reactions.^[^ ^32^, ^33^, ^34^ ^]^

The development of micro‐architected electrode materials unlocks new possibilities for fully 3D batteries, a concept that originated over two decades ago. Moving beyond conventional 2D layered batteries, various 3D battery architectures have been explored, as discussed by Long et al. in their review article from 2004.^[^ ^35^ ^]^ They outlined four representative designs: interdigitated cathode and anode cylindrical arrays, interdigitated electrode plate arrays, cylindrical arrays of one electrode with in‐filled counter electrode, and aperiodic porous electrode with in‐filled counter electrode. The last design, requiring the deposition of a conformal solid electrolyte layer on all surfaces of one electrode and a dense counter electrode in‐fill into micro or nanopores, is yet to be realized with existing manufacturing techniques. State‐of‐the‐art reports are limited to the fabrication of a single electrode with aperiodic porous structures.^[^ ^36^ ^]^ Semiconductor‐based processes have been successfully utilized to fabricate 3D batteries with arrays of 100 µm‐diameter Si pillars as anodes and in‐filled cathode slurries; the anode material in these constructs is limited to silicon, notorious for its enormous volume expansion upon lithiation.^[^ ^37^ ^]^. Attempts to fabricate other materials in this rod‐array geometry have had limited success.^[^ ^38^, ^39^ ^]^ 3D batteries with the interdigitated‐plate configurations have been successfully created through DIW, with both cathode and anode inks extruded onto a substrate to form stacked filaments, achieving up to 16 layers and giving a maximum height‐to‐thickness ratio of 8:1 for the electrode plates.^[^ ^3^, ^4^ ^]^ Stacking the layers higher remains challenging because the viscoelastic nature of the inks leads to layer collapse. Advancements in VP‐based AM techniques have expanded the flexibility in attainable 3D electrode geometries, allowing for more intricate micro‐scale structures, for example, horizontally oriented beams with ≈50 µm diameters. The AM method to create architected electrodes described in this work enables a new free‐standing 3D interdigitated LIB design where both electrodes are structured as interdigitated plates, composed of beams ranging from 50 to 70 µm in diameter, as depicted in Figure 6b. The carbon anode is produced following a VP 3D printing and pyrolysis method introduced by Narita et al., while the LCO cathode was fabricated through the AM technique demonstrated in this work, both according to similar yet complementary CAD models (Figure 6b(i–iii)).^[^ ^40^ ^]^ The surface of the LCO cathode is coated with a photopolymerizable gel polymer electrolyte resin, which also fills the free volume in each plate via capillary action, followed by UV curing. Subsequently, the carbon anode is integrated, with additional electrolyte resin filling any remaining gap space between the electrodes prior to the final cure. Figure 6b(iv) illustrates a schematic of the fully assembled 3D battery, with Figure 6b(v) containing an optical image of a fabricated sample. A significant challenge in this design is ensuring the resin thoroughly permeates the empty volume in the plates and forms a uniform layer on their surfaces, without overfilling the interspace between adjacent LCO electrode plates, requiring precise viscosity control of the electrolyte resin. Despite this challenge, the free‐standing 3D interdigitated batteries, with their high active material loading and minimized ion transport distances, show promise in breaking the conventional compromise between energy density and power density in energy storage technologies.^[^ ^39^, ^40^ ^]^

## 6 Conclusion

We developed a high‐resolution gel infusion additive manufacturing process for creating 3D micro‐architected cathode materials, with LiCoO_2_ as a model material system. Leveraging on vat photopolymerization 3D printing, this approach overcomes the inherent limitations in resolution of existing fabrication methods, enabling the production of free‐standing and binder‐free micro‐architected LCO electrodes with large freedom in structural design. The micro‐sized beam diameters of 45 µm allow for efficient Li‐ion transport, and the tunable LCO microstructure brings about enhanced mechanical strength. This versatile technique eliminates the need for extensive photoresin design and printing parameter optimization to create architected cathode materials with high resolution, and only requires the change of target material precursors in the infusion solution, which is demonstrated by the fabrication of 3D micro‐architected LiNi_0.33_Mn_0.33_Co_0.33_O_2_ lattices with 45 µm beam diameters using the same process.

## 7 Experimental Section

### Photoresin Preparation

57.5 g (0.10 mol) of poly(ethylene glycol) diacrylate *M*_n_ = 575 (Sigma–Aldrich) was dissolved in 40 mL *N,N*‐dimethylformamide (DMF) (⩾99.8%, Sigma–Aldrich). 506.1 mg (1.33 mmol) of 2‐dimethylamino‐2‐(4‐methyl‐benzyl)‐1‐(4‐morpholin‐4‐yl‐phenyl)‐butan‐1‐one (Omnirad 379; IGM Resins), 335.4 mg (1.25 mmol) of Bis[4‐(dimethylamino)phenyl]methanone (Michler's ketone; 98%, Sigma–Aldrich) and 14.90 mg (0.06 mmol) of 1‐(phenyldiazenyl)naphthalen‐2‐ol (Sudan I; ⩾95%, Sigma–Aldrich) were dissolved in 11.3 mL of DMF separately, and then mixed with the previous solution to form a clear photoresin with orange color. The photoresin was stored away from light before used for printing.

### 3D Printing and Post‐Printing Process

3D models of all the architectures were created using a computer‐aided design (CAD) software (Blender, Blender Foundation) and printed with a 385 nm wavelength digital light processing (DLP) 3D printer (Titan 3, MicroSLA). The octet truss lattice structure was designed with 120 µm beam diameter and 1000 µm unit cell size. The overall geometry was designed to be circular to fit the coin cell, with 12.8 mm in diameter and 4.5 mm in thickness. The printing parameters can be found in Table S2 (Supporting Information). The printed organogel structures were soaked in DMF at room temperature to remove the unreacted photoresin components and then in deionized water at 80 °C for 2 h to allow solvent exchange. The hydrogel microlattices were infused in a 2 M LiNO_3_ (Sigma–Aldrich) and Co(NO_3_)_2_ (Sigma–Aldrich) aqueous solution at 80 °C for 48 h. The heating temperature started with a ramp to 400 °C at 0.25 °C min^−1^, followed by a 5 h isothermal hold and then a ramp to 700 °C, 800 or 900 °C at 2 °C min^−1^, before cooling to room temperature at 1 °C min^−1^. The calcination took place under a compressed air flow of 50 mL min^−1^ at a pressure of approximately 0.01 atm by the end of the isothermal hold at 400 °C, and the atmosphere was maintained or switched to an Ar flow of 200 mL min^−1^ at a pressure of approximately 1 atm before the ramp to peak temperatures.

### Characterizations of the Micro‐Architected Ion‐Infused Hydrogel

Thermogravimetry analysis (TGA; TGA 550, TA Instruments) and differential scanning calorimetry (DSC; DSC 25, TA Instruments) were conducted for the hydrogel microlattice infused with lithium and cobalt nitrates, both heated in an air flow at 25 mL min^−1^. TGA temperature ramps at 20 °C min^−1^ from room temperature to 120 °C, followed by an isothermal hold for 60 min for complete water removal, and then ramps at 2 °C min^−1^ to 700 °C. DSC temperature ramps at 1 °C min^−1^ from 25 to 400 °C.

### Characterizations of the Micro‐Architected LiCoO_2_

LCO microlattices were imaged by a scanning electron microscope (SEM; Versa 3D DualBeam, Thermo Fisher) at an accelerating voltage of 10 kV. Energy‐dispersive X‐ray spectroscopy (EDS; Quantax 200, Bruker) elemental analysis was performed in the same instrument with the same voltage. Gallium focused ion beam (FIB) milling was performed in the same instrument to mill cross‐sections using an accelerating voltage of 30 kV and a current of 65 nA. A series of FIB cleaning was performed using an accelerating voltage of 30 kV and currents of 30, 15, 7, and 3 nA. The ion channeling images were taken at accelerating voltage of 30 kV and a current of 10 pA. LCO microlattices were pulverized using a mortar and pestle before analyzed by a powder X‐ray diffractometer (XRD; SmartLab, Rigaku) with a Cu K^α^ source at 40 kV and 50 mA. The amount of lithium and cobalt atoms in LCO microlattices were analyzed using inductively coupled plasma mass spectroscopy (ICP‐MS; 8800 Triple Quad ICP‐MS, Agilent). About 25 mg of the LCO microlattice was digested in about 6 mL of 70% nitric acid at 90 °C with reflux heating for 12 h and diluted with 5% nitric acid to 50 mL, and then further diluted twice in 5% nitric acid to reach x400 dilution. Eight external standards were prepared with lithium and cobalt standard solutions for ICP through serial dilution with 5% nitric acid to generate calibration curves. Quasistatic uniaxial compression tests for LCO microlattices were conducted using a dynamic mechanical analyzer (DMA; DMA 850, TA Instruments) with a 15 mm diameter compression clamp at a strain rate of 10^−3^ s^−1^.

### Nanoindentation and Electron Backscatter Diffraction (EBSD)

LCO microlattices were mounted in epoxy (FibreGlast 2000) in a silicone mold and fully cured at 70 °C for 24 h, and then progressively polished with 320 grit, 400 grit, 600 grit, 800 grit and 1200 grit silicon carbide papers (Buehler) and subsequently 9, 6, 3, 1 µm and 50 nm grit monocrystalline diamond suspensions (Buehler). The in situ nanoindentation experiments were performed in SEM (Versa 3D DualBeam, Thermo Fisher) using a nanomechanical testing system (FT‐NMT04, FemtoTools AG) under displacement‐controlled mode using a diamond Berkovich tip with a micro‐electromechanical system (MEMS)‐based capacitive force sensor (FT‐S200000). Each indentation was executed at a displacement rate of 5 nm s^−1^ up to a maximum depth of 150 nm, and indentation arrays had a spacing of 1.5 µm between individual indents. Continuous stiffness measurement (CSM) technique was utilized, which superimposed a 200 Hz oscillating displacement of 3 nm in amplitude to quasi‐continuously measure contact stiffness as a function of depth during loading, which was calculated as

(1) $$S=\frac{a_{f}}{a_{d}}cos\varphi$$

where *a*_*d*_ is the displacement amplitude and *a*_*f*_ is the force amplitude of the CSM oscillation as functions of contact depth, and φ is the phase angle between them. The reduced modulus of the sample‐indenter combination (*E*_*r*_) as a function of contact depth was calculated as

(2) $$\frac{1}{E_{r}}=\frac{\sqrt{\pi }}{2\beta }\frac{S}{\sqrt{A}}$$

where the geometric factor β = 1.034 was chosen for the Berkovich tip, and *A* is the contact area as a function of contact depth *h*_*c*_, obtained from tip calibration and fitted using an area function with seven coefficients *C*_1_ through *C*_7_

(3) $$A\left(h_{c}\right)=24.5h_{c}^{2}+\sum_{n=1}^{7}C_{n}n^{2}dh_{c}e^{-nh_{c}/d}$$

where the fitting constant *d* = 0.3 was chosen for the tip. The indentation modulus of the sample (*E*_*s*_) as a function of contact depth was calculated as

(4) $$\frac{1}{E_{r}}=\frac{1-\nu_{s}^{2}}{E_{s}}+\frac{1-\nu_{i}^{2}}{E_{i}}$$

assuming the Poisson's ratio ν_*s*_ = 0.3 for LCO, and the Young's modulus and Poisson's ratio of the diamond indenter tip to be *E*_*i*_ = 1140 GPa and ν_*i*_ = 0.07, respectively. Hardness *H* as a function of contact depth was calculated as

(5) $$H=\frac{P(h_{c})}{A(h_{c})}$$

where *P* is the load as a function of contact depth. For all experiments, the indentation modulus and hardness were extracted by averaging the values at the depth of 40–41 nm to eliminate surface effects. An EBSD system (AZtec, Oxford Instruments) in SEM (LEO 1550 VP, Zeiss) was used, with a 120 µm aperture at 20 kV, to identify the crystal orientations of LCO grains on which the nanoindentation experiments were conducted. Data analysis was done in AZtecHKL software, and EBSD maps display the inverse pole figure in the z direction, which aligns with the indentation direction.

### Cyclic Voltammetry (CV) and Electrochemical Cycling

The slurry‐based electrodes were used for CV analysis. The LCO microlattices were pulverized into a fine powder using a pestle and mortar. To make the electrode slurry, 255 mg of the pulverized LCO powder was thoroughly mixed with 15 mg of Super C65 (MTI), 30 mg of polyvinylidene fluoride (PVDF, average M_w_ ≈534000, Sigma–Aldrich) and 0.5 mL of N‐methyl‐2‐pyrrolidone (NMP; anhydrous, 99.5%, Sigma–Aldrich) in a plastic vial using zirconia ball milling at 1000 rpm in a vortex mixer (LP Vortex Mixer, Thermo Scientific). The slurry paste was coated on a 20 µm thick aluminum foil using a micrometer film applicator (SH0335, TQC Sheen) and dried in a vacuum oven at 50 °C for 24 h to obtain an electrode film with 20 µm thick dry slurry coating. Electrode disks of 9.5 mm in diameter were punched out of the film and dried in a vacuum oven at 100 °C for 24 h before transferred to an Ar‐filled glovebox (HE‐243‐XW, Vacuum Atmospheres). The slurry electrode was assembled in a CR2032 coin cell (MTI) against a lithium foil (99.9%, Sigma–Aldrich) counter electrode with a 25 µm thick polypropylene‐polyethylene‐polypropylene trilayer microporous separator (Celgard 2325), a wave spring, and a flooded amount of 1 M lithium hexafluorophosphate (LiPF_6_) in ethylene carbonate (EC) and diethyl carbonate (DEC) with 1:1 (v/v) ratio (Sigma–Aldrich). The coin cell assembly was conducted using a hydraulic crimper (TOB‐MR‐120, TOB New Energy) by applying 800 psi on the coin cell. CV was performed at a scanning rate of 0.05 mV s^−2^ between 3.0 and 4.2 V using a battery cycling system (BCS‐805, BioLogic). The LCO microlattice with an overall height of 1.80 mm was assembled in a CR2032 coin cell with the same lithium counter electrode, separator and electrolyte as the slurry electrode, with an additional 1.78 mm thick high density polyethylene (HDPE) washer to centrally position the microlattice and support the compressive stress from coin cell assembly. The schematics of the components of the coin cell are illustrated in Figure 5b. Electrochemical cycling tests of the LCO microlattice coin cells were conducted using a battery cycling system (BTS4000, Neware) between 3.0 and 4.2 V at C‐rates of C/40 (3.5 mA g^−1^), C/20 (7 mA g^−1^), C/10 (14 mA g^−1^), C/5 (28 mA g^−1^), C/2 (70 mA g^−1^), 1C (140 mA g^−1^), and 2C (280 mA g^−1^), calculated based on a 140 mAh g^−1^ practical capacity of LCO.

## Conflict of Interest

The authors declare no conflict of interest.

## Supporting information

Supporting Information

## Data Availability

The data that support the findings of this study are available from the corresponding author upon reasonable request.
