# Supplementary material for: Micro‐Architected Lithium Cobalt Oxide
Source: Adv Sci (Weinh). 2025 Oct 8;12(43):e13312. doi: 10.1002/advs.202513312 (PMC12631914; doi:10.1002/advs.202513312)
Supplement: Supplementary file 1 — Supporting Information [file ADVS-12-e13312-s001.pdf]

Supporting Information

N,N-Dimethylformamide	51.3 mL
Poly(ethylene glycol) diacrylate (average M_n 575)	0.10 mol
Omnirad 379	1.33 mmol
Michler's ketone	1.25 mmol
Sudan I	0.06 mmol

Table S1. Composition of the customized arylate-based photoresin for VP 3D printing.

Slice thickness (mm)	0.01	LED Current (5.86 mA per unit)	300		
	Exposure Time (s)	Lift Height (mm)	Lift Speed (mm min^{-1})	Down Speed (mm min^{-1})	Delay Time (s)
First layer	3	7	10	150	2
All rest layers	0.8	7	25	150	2

Table S2. Printing parameters for the customized photoresin using Titan 3 DLP printer.

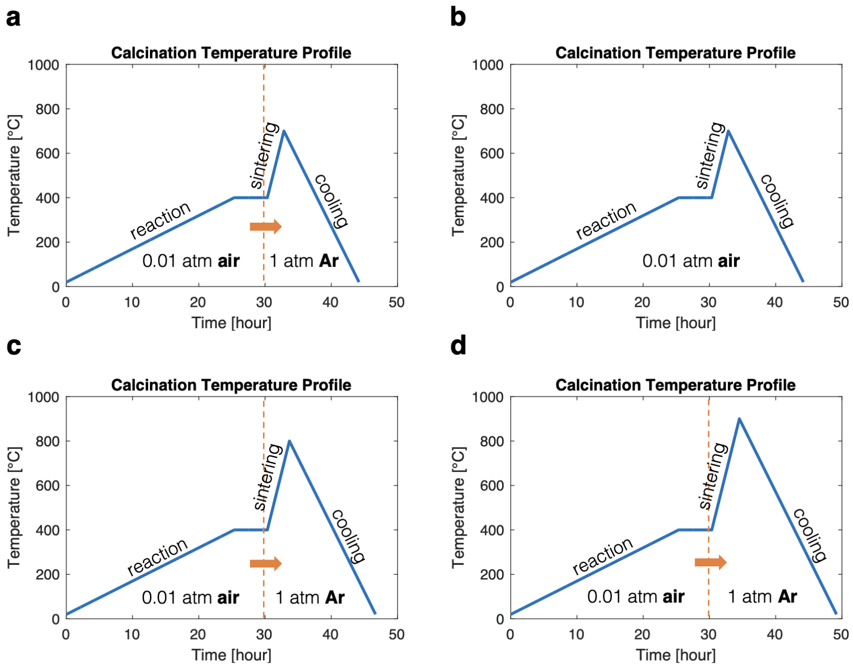

Figure S1. Various LCO calcination temperature profiles with different sintering pressures and gas species: a) under 1 atm Ar at 700 °C; b) under 0.01 atm air at 700 °C; c) under 1 atm Ar at 800 °C; d) under 1 atm Ar at 900 °C.

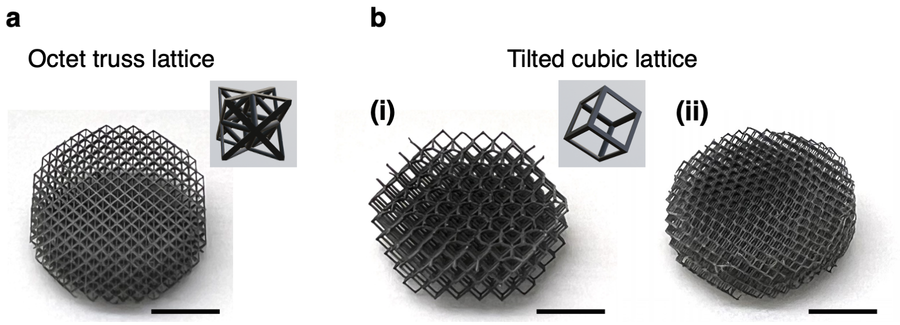

Figure S2. Optical images of 3D micro-architected LCO lattices with a) octet truss unit cell and b) tilted cubic unit cell in varies sizes. Insets show the octet truss and tilted cubic unit cells. Scale bars are 2 mm for (a), (b(i) and (ii)).

Sample mass (mg)	23.12
Li mass concentration in diluted sample solution measured by ICP-MS (μg L^{-1})	78.51 ± 1.01
Co mass concentration in diluted sample solution measured by ICP-MS (μg L^{-1})	661.49 ± 6.87
Li mass in sample (mg)	1.57 ± 0.02
Co mass in sample (mg)	13.23 ± 0.14
Theoretical O mass in sample, assuming electroneutrality (mg)	7.20
LCO mass in sample	22.00
LCO mass percentage	95.1%
**Carbon mass percentage**	**4.9%**
**Li:Co molar ratio**	**1.01:1**

Table S3. Carbon mass percentage and Li:Co molar ratio of the micro-architected LCO sample sintered under 1 atm Ar at 700 °C measured by ICP-MS.

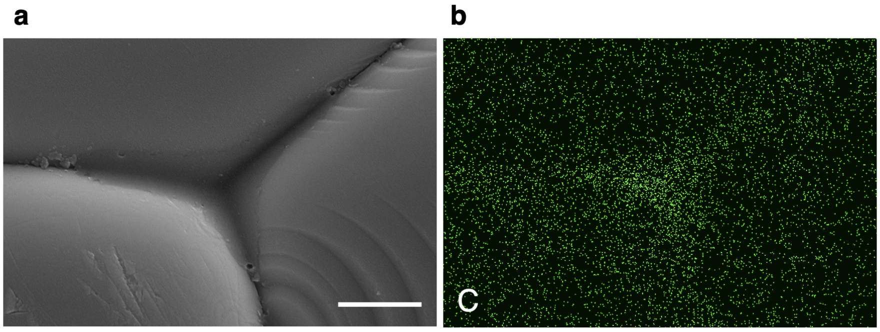

Figure S3. a) SEM image and b) the corresponding EDS carbon element mapping showing carbon at LCO grain boundaries, from a sample sintered under 1 atm Ar at 900 °C. The scale bar is 1 µm.

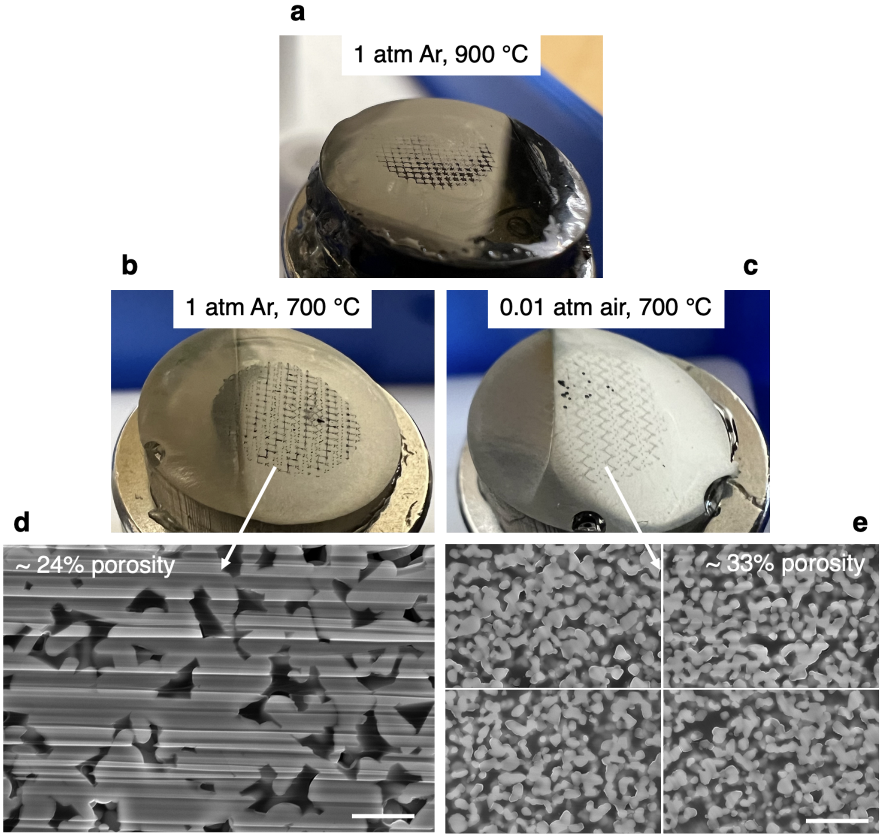

Figure S4. Optical images of 3D micro-architected LCO lattices mounted in epoxy and after progressive polishing to a 50 nm grit suspension, with samples sintered a) under 1 atm Ar at 900 °C, b) under 1 atm Ar at 700 °C and c) under 0.01 atm air at 700 °C. SEM images of the cross-sections of epoxy mounted LCO lattices sintered d) under 1 atm Ar at 700 °C and e) under 0.01 atm air at 700 °C, after mechanical and FIB polishing, for porosity estimation. The thorough penetration of epoxy into the porous volume within LCO indicates interconnected pore spaces, allowing for the flow of liquid electrolyte and gel polymer electrolyte resin. Scale bars are 2 μm for (d) and (e).

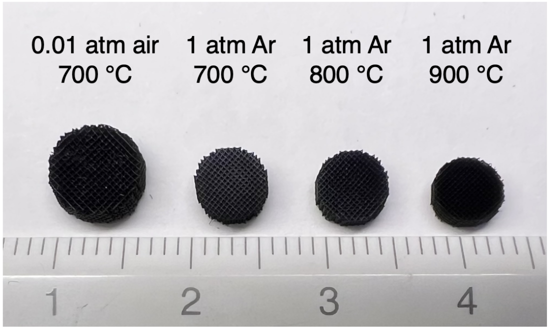

Figure S5. Optical image of 3D micro-architected LCO lattices sintered under different atmospheres at various maximum temperatures.

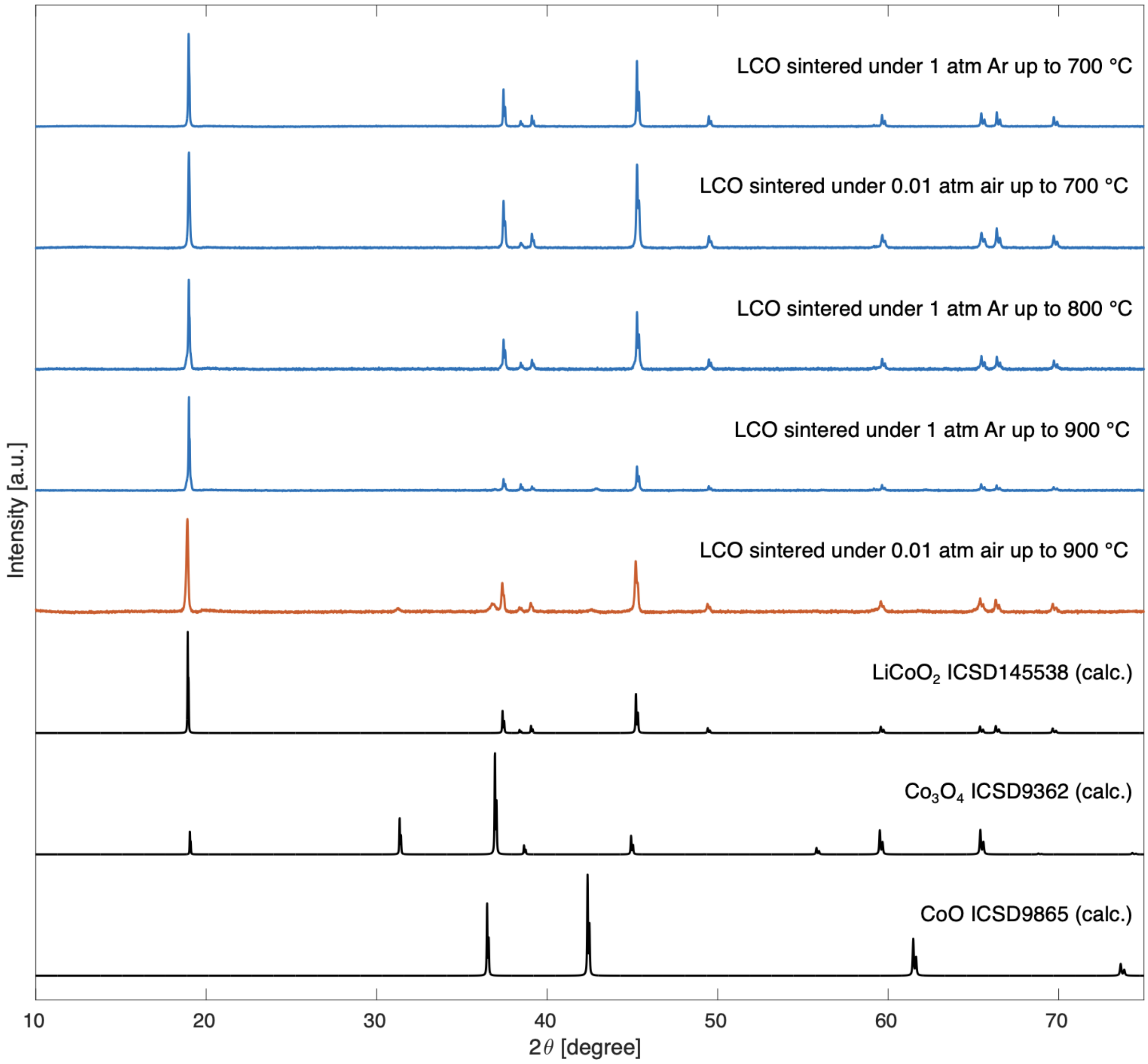

Figure S6. XRD patterns for the additively manufactured LCO sintered under different pressures and gas species at various maximum temperatures, with references from ICSD.

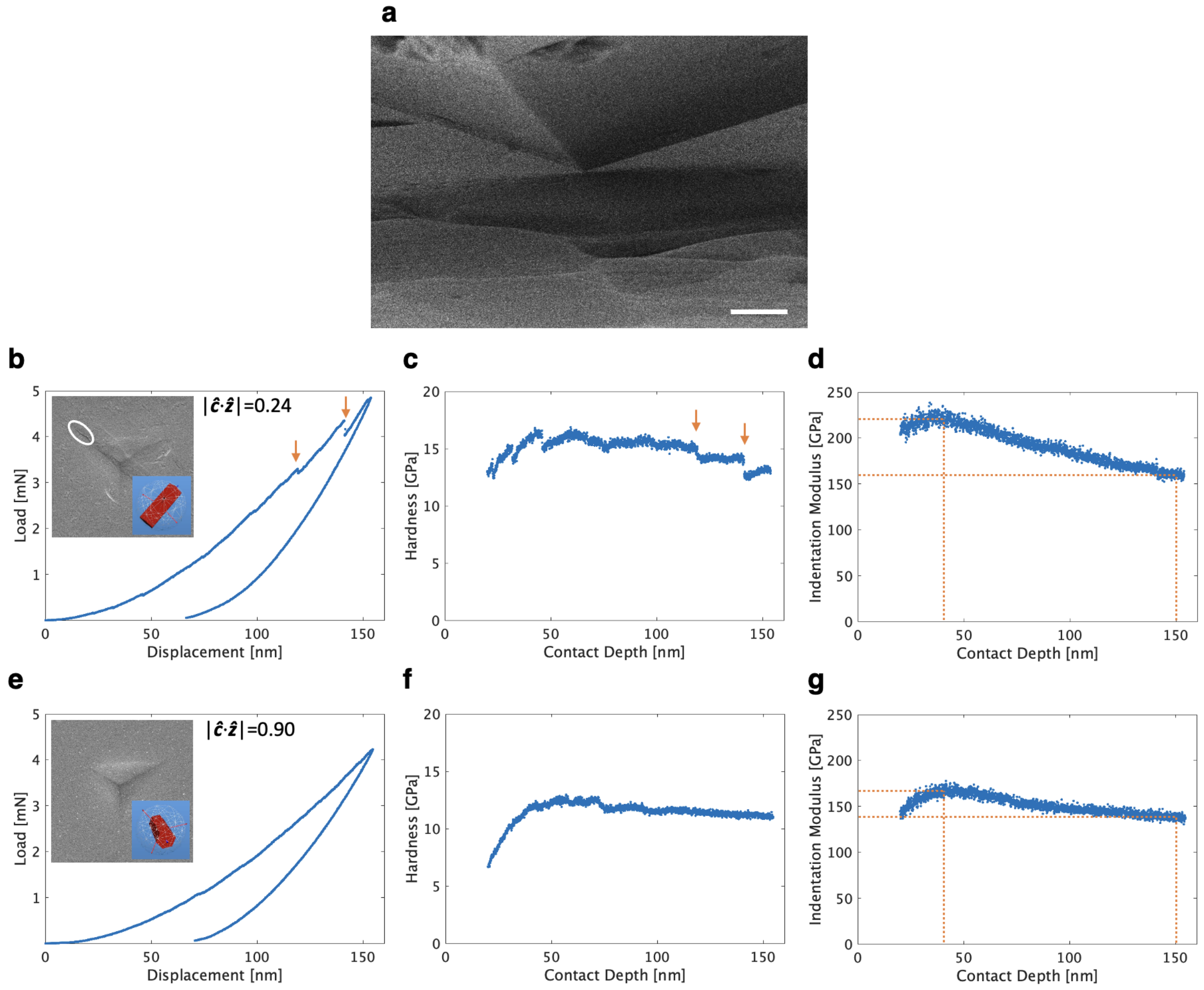

Figure S7. SEM image of the in situ nanoindentation experiment on LCO using a diamond Berkovich tip. b) Load–displacement curve and the corresponding c) hardness and d) indentation modulus during loading for a representative indentation with orientation nearly perpendicular to [0001] of LCO crystal ($|\hat{\boldsymbol{c}} \cdot \hat{\boldsymbol{z}}| = 0.24$). e) Load–displacement curve and the corresponding f) hardness and g) indentation modulus during loading for a representative indentation with orientation nearly aligned along [0001] of LCO crystal ($|\hat{\boldsymbol{c}} \cdot \hat{\boldsymbol{z}}| = 0.90$). The scale bar is 3 μm for (a).

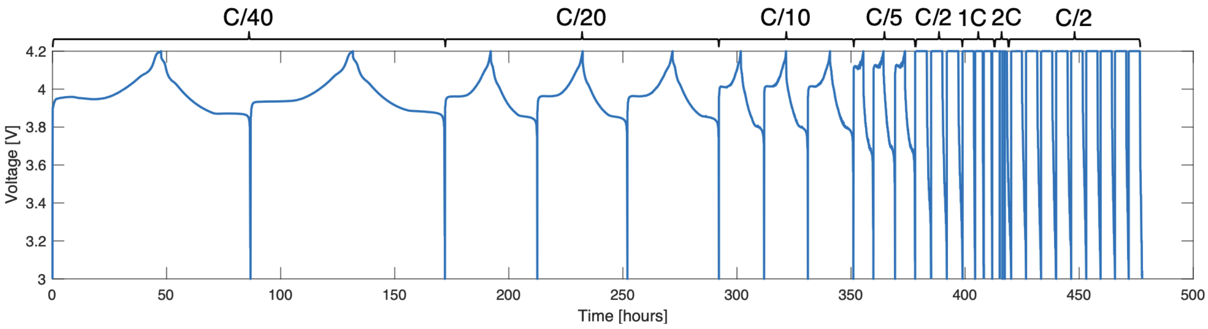

Figure S8. Potential curves of the LCO microlattice cycled between 3.0 and 4.2 V at different C-rates.

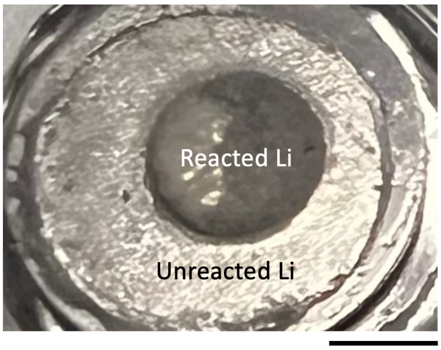

Figure S9. Optical image of Li metal anode after full cell cycling. The scale bar is 5 mm.

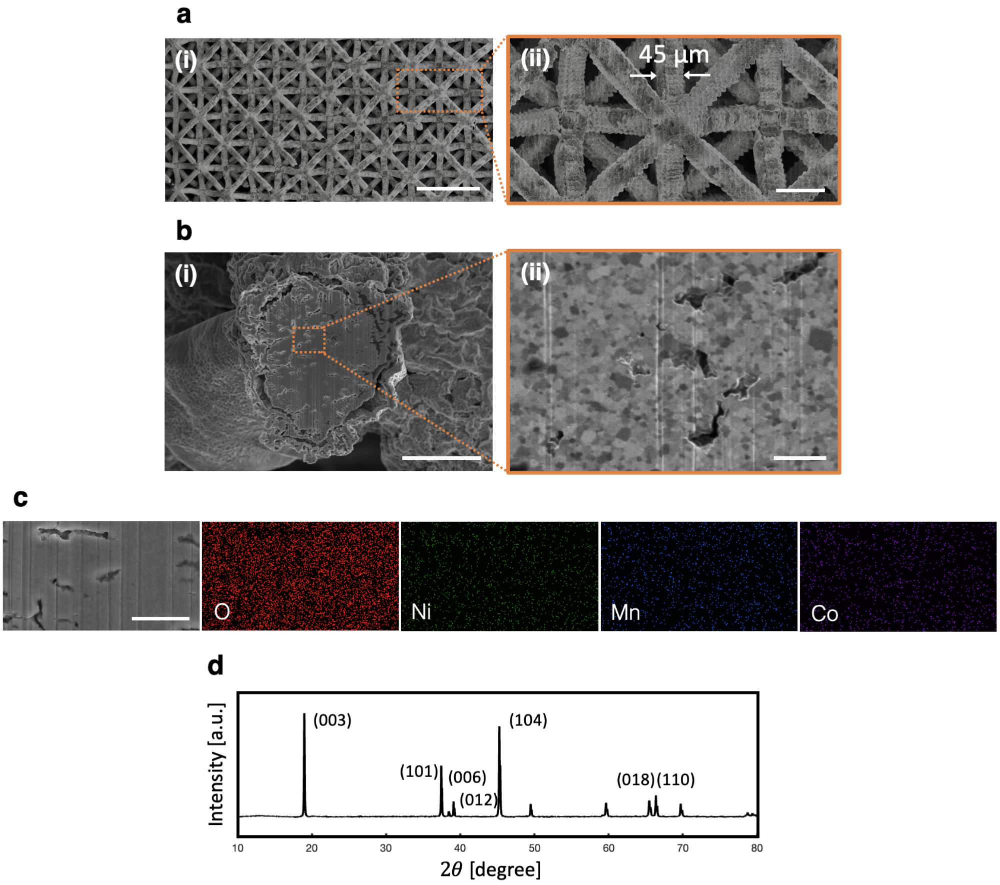

Figure S10. a) SEM images of a 3D micro-architected NMC111 octet truss lattice in top view. b) SEM and ion channeling contrast images, and c) EDS element maps of its beam cross-section. d) XRD pattern for the additively manufactured NMC111 sintered under 1 atm Ar at 700 °C. Scale bars are 500 µm for (a(i)), 100 µm for (a(ii)), 20 µm for (b(i)), 1 µm for (b(ii)) and 2 µm for (c).
